# Correction

**DOI:** 10.1080/22221751.2024.2355702

**Published:** 2024-06-02

**Article title:** A one-year follow-up study of systematic impact of long COVID symptoms among patients post SARS-CoV-2 omicron variants infection in Shanghai, China

**Authors:** Cai J, Lin K, Zhang H, Xue, Q, Zhu, K, Yuan G, Sun Y, Zhu F, Ai J, Wang S, and Zhang W.

**Journal:** *Emerging Microbes & Infections*

**DOI***:* https://doi.org/10.1080/22221751.2023.2220578

When this article was first published online the asterisk which should have been linked to the footnote about being co-first authors was missing for the first three authors. That is, Cai J, Lin K, and Zhang H have contributed equally to this work and share first authorship.

Other errors include the word “patients” in Figure 1 being incorrectly spelled as “parients”, the word "Skin rash” in Figure 3 being mistakenly spelled as “Skinrash”, the word "Brain fog” in Figure 3 is mistakenly spelled as “Brain frog”, the word "Abdominal pain” in Figure 3 is missing the word "pain". These have been corrected in the original article.

The authors states that these do not change the scientific conclusions of the article in any way.

